# Long pedunculated tumor from the ureteral stump protruding into the bladder after nephrectomy: a case report

**DOI:** 10.1007/s12672-026-05028-7

**Published:** 2026-05-07

**Authors:** Jiahao Li, Jintao Guo, Jianbiao Huang, Qi Wu, Ji Huang

**Affiliations:** Jiangxi Cancer Hospital, The Second Affiliated Hospital of Nanchang Medical College, Jiangxi Clinical Research Center for Cancer, Nanchang, Jiangxi, China

**Keywords:** Renal cell carcinoma, Nephrectomy, Carcinoma, Transitional cell, Ureteral stump

## Abstract

The ureteral stump carcinoma after radical nephrectomy is exceedingly rare. We report a 75-year-old woman who underwent right laparoscopic nephrectomy in April 2024 for a renal mass detected during hypertension evaluation. Pathology confirmed clear cell RCC, ISUP grade 2, with negative ureteral margins. In December 2025, during cystoscopy for contralateral renal calculus management, A long pedunculated tumor, arising from the ureteral stump and protruding into the bladder, was incidentally discovered at the right ureteral orifice. Biopsy revealed low-grade non‑invasive TCC. Subsequent laparoscopic partial cystectomy and ureterectomy confirmed ureteral non‑invasive low‑grade TCC originating from the stump. This case highlights that even with initial negative margins, metachronous primary tumors may arise in the urinary tract, necessitating reconsideration of ureteral stump management after nephrectomy.

**Supplementary Information:**

The online version contains supplementary material available at 10.1007/s12672-026-05028-7.

## Introduction

metachronous multiple primary malignant neoplasms (MPMNs) refer to newly developed, histologically distinct, independent tumors occurring after an initial primary malignancy. Patients with genitourinary malignancies often develop metachronous primary tumors. Individuals with prostate cancer, renal cancer, and renal pelvic cancer have an elevated risk for metachronous primary tumors, frequently arising in the colon, lungs, and bladder—a group strongly associated with smoking [1].

We report a case of a long pedunculated tumor from the ureteral stump protruding into the bladder. To our knowledge, this is the first domestic report of Pedunculated tumor of ureteral stump protruding into bladder. This report describes a case of MPMNs within the urinary system. The case demonstrates the importance of clinical awareness and provides valuable insights to guide long-term monitoring strategies, ultimately aiming to improve patient outcomes.

## Case report

**Fig. 1:**
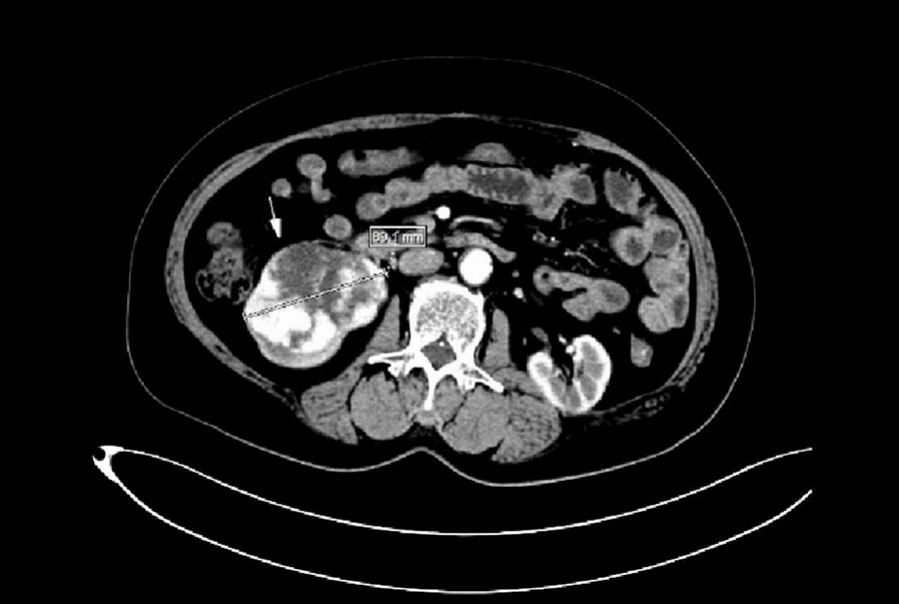
CT scan reveals an 8.9 cm × 6.2 cm mass in the lower pole of the right kidney involving the renal pelvis

A 75-year-old female presented with a history of hypertension. Ultrasound of the urinary tract revealed a space-occupying lesion in the right kidney. Physical examination showed no remarkable positive findings. The patient had a medical history of hypertension for more than ten years, with no smoking history or long-term medication use. Contrast-enhanced CT of the kidneys demonstrated a mixed-density mass measuring approximately 8.9 cm × 6.2 cm in the lower pole of the right kidney, involving the renal pelvis (Figure 1). The enhancement pattern was characterized by rapid wash-in and wash-out, suggestive of renal cell carcinoma.

**Fig. 2:**
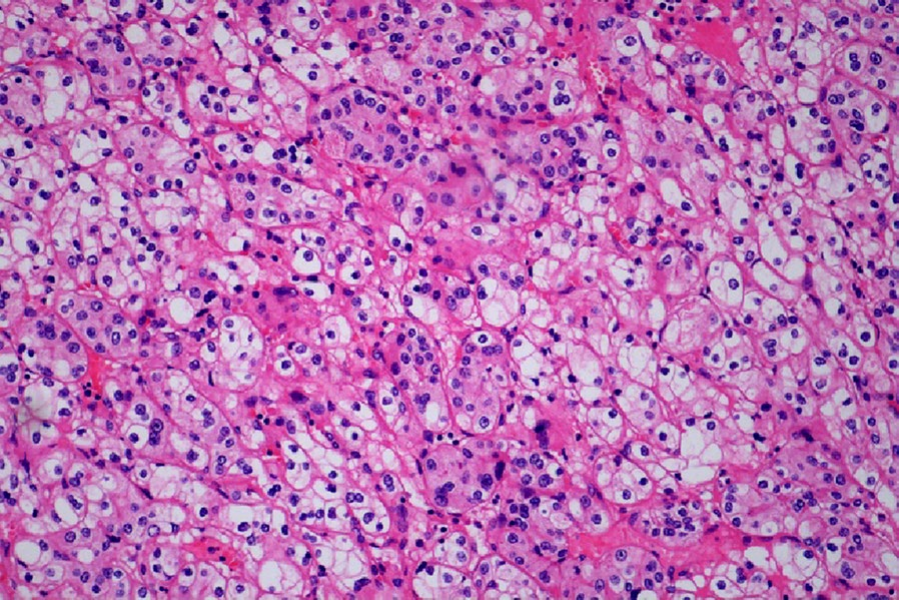
Histopathological examination (H&E stain, 10 × 40 magnification) of the kidney specimen confirms clear cell renal cell carcinoma

On April 23, 2024, the patient underwent a laparoscopic radical right nephrectomy. The resected specimen was sent for pathological evaluation, which confirmed clear cell carcinoma, ISUP grade 2, with negative ureteral margins (Figure 2). Follow-up CT scans performed in March and September 2025 showed postoperative changes without evidence of recurrence.

**Fig. 3:**
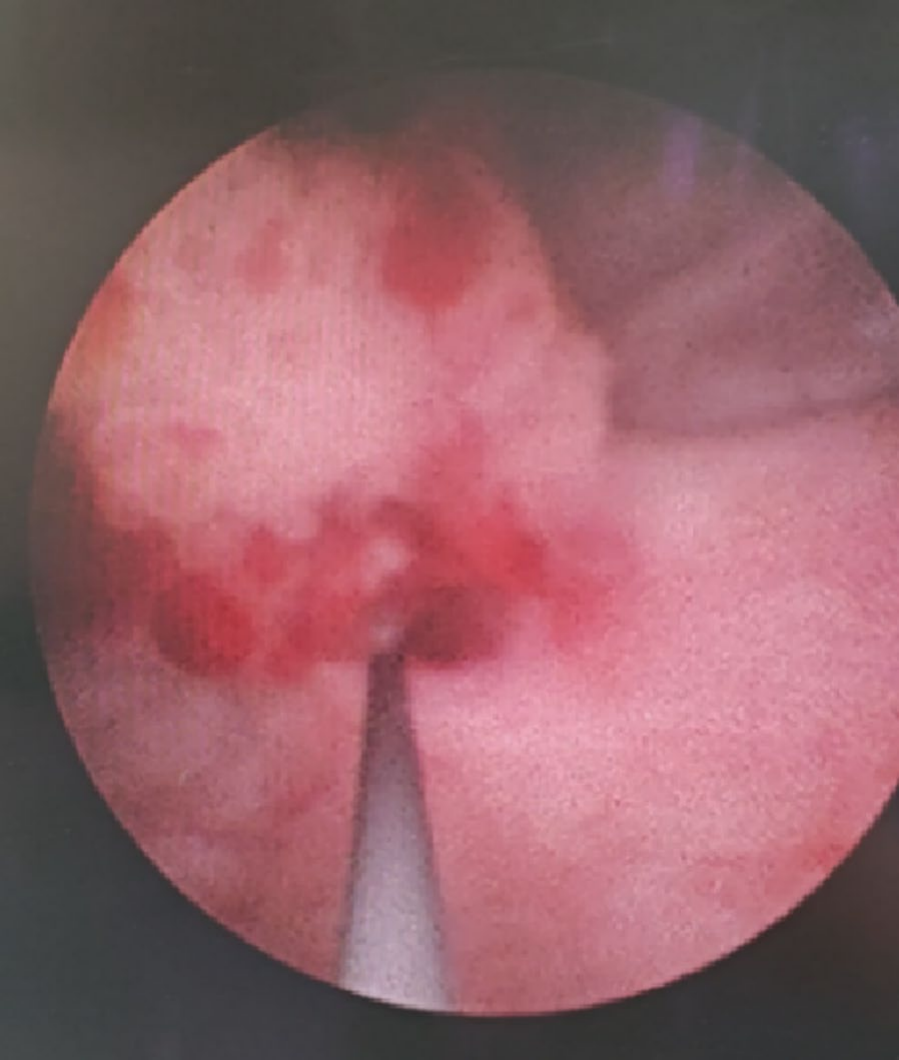
Intraoperative photograph showing the ureter tumor protruding into the bladder

**Fig. 4:**
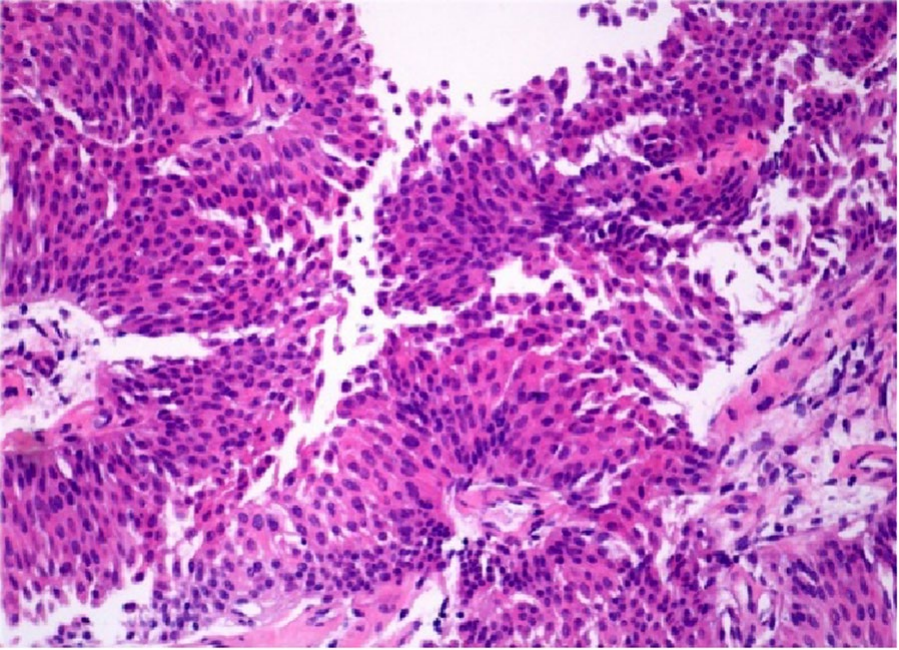
Histopathological examination (H&E stain, 10 × 40 magnification) of the bladder specimen

**Fig. 5:**
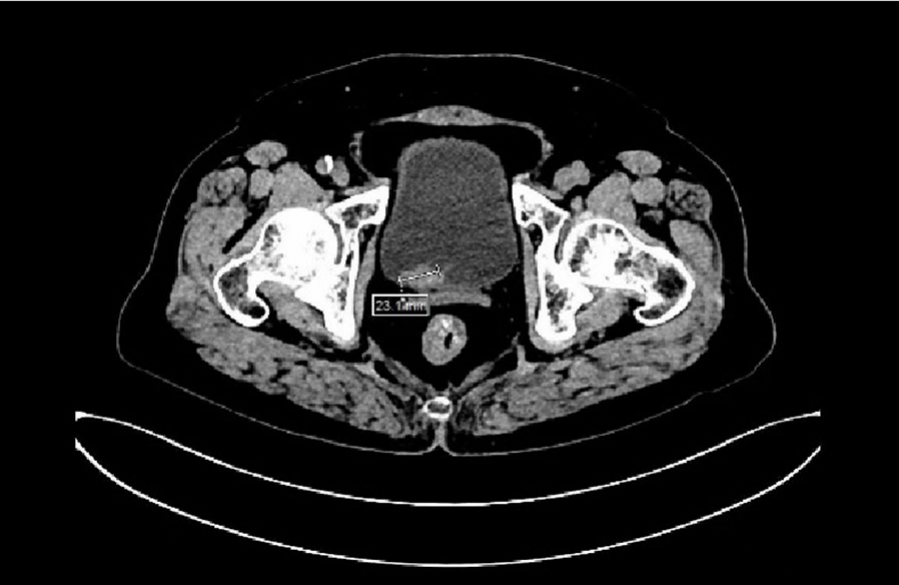
Preoperative CT scan revealing a 2.3 cm × 1.3 cm nodular shadow within the bladder

In December 2025, the patient was readmitted for management of a left lower calyceal renal calculus. To relieve associated hydronephrosis, a ureteral stent was placed under direct cystoscopic guidance. During the procedure, an unexpected cauliflower-like neoplasm protruding from the ureteral lumen was observed at the right ureteral orifice (Figure 3). A biopsy was taken with forceps, and pathological analysis revealed low-grade non-invasive transitional cell carcinoma (Figure 4). Preoperative CT imaging identified a new nodule on the posterior wall of the right side of the bladder, measuring approximately 2.3 cm × 1.3 cm, while the right ureter appeared unremarkable (Figure 5).

**Fig. 6:**
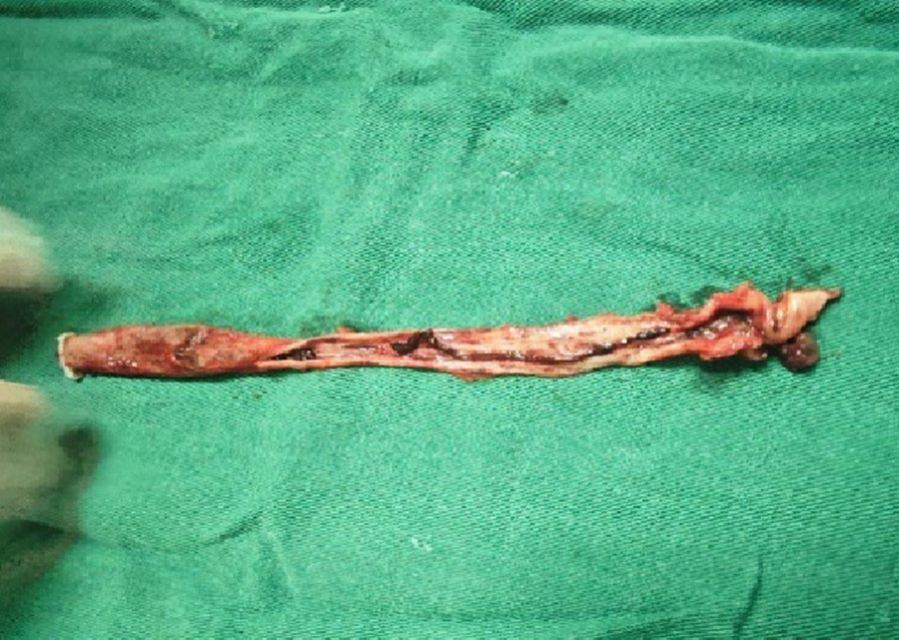
Gross photograph of the completely resected ureter with the attached tumor

**Fig. 7:**
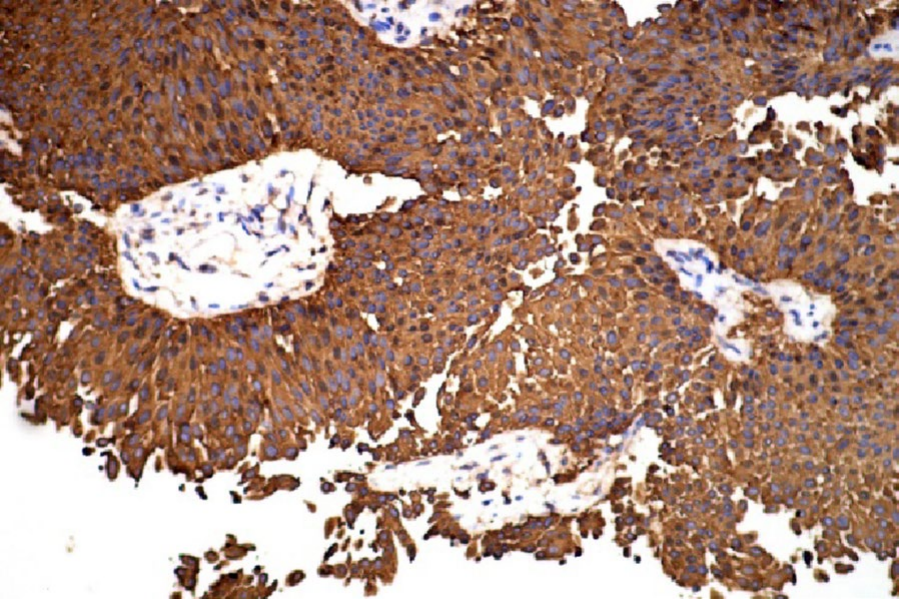
Immunohistochemical staining for CK7 on the postoperative ureteral specimen confirmed the diagnosis of transitional cell carcinoma

On December 25, 2025, the patient underwent surgery under general anesthesia. The ureteral tumor seen during the operation showed a smooth and slender pedicle structure, the pedicle was about 8 cm long, and the distal end of the cauliflower like tumor protruded into the bladder cavity (Figure 6). Ureterectomy with partial cystectomy was performed. Histopathological examination of the resected ureter confirmed non-invasive low-grade transitional cell carcinoma (Figure 7), consistent with the earlier biopsy findings. The patient recovered well postoperatively and is scheduled for follow-up with an abdominal CT and cystoscopy at three months, with subsequent annual reviews thereafter.

## Discussion

While some cases involve synchronous tumors—such as renal cell carcinoma occurring with bladder transitional cell carcinoma [2], or the simultaneous presence of both renal cell carcinoma and transitional cell carcinoma components within the same kidney [3]—the present case is distinct. No ureteral abnormalities were detected during the initial surgery, and the subsequent ureteral tumor exhibited a unique morphology.

Previous reports have indicated that hematuria is the predominant symptom of ureteral stump cancer after nephrectomy, with tumors typically presenting as a mass-like lesion [4, 5]. In contrast to these common mass-like tumors, our intraoperative findings revealed a smooth-surfaced, slender, cord-like tumor within the ureter, which extended into the bladder where a cauliflower-like mass was visible. Meanwhile, the patient did not present with hematuria. We hypothesize that this long, pedunculated morphology contributed to the absence of symptoms and the difficulty in detection by imaging, potentially leading to the diagnostic delay observed in this case [6] .The presence of occult lesions and the timing of diagnosis are important considerations, as microscopically undetectable cancerous foci or carcinoma in situ may have been present at the initial surgery and subsequently progressed to clinically detectable tumors postoperatively. Due to anatomical constraints or the nature of the primary tumor, a complete nephroureterectomy may not always be feasible. Therefore, excising the ureter as distally as technically feasible during nephrectomy for renal masses is advisable.

The long pedunculated tumor within the ureter observed intraoperatively bears a high morphological resemblance to a benign fibroepithelial polyp (FEP), which can also present as a snake-like or cord-like structure protruding into the bladder [7]. Another reported case described a patient with simultaneous renal cell carcinoma, bladder transitional cell carcinoma, and a ureteral FEP [8]. Differentiation primarily relies on careful endoscopic observation: FEPs typically have a smooth surface, regular vascular pattern, and a narrow base, whereas transitional cell carcinomas often appear papillary or cauliflower-like, with surface necrosis, disorganized vasculature, and a broad base or infiltrative appearance.

This suggests that the local anatomical and physiological environment resulting from the surgery itself (e.g., local ischemia or chronic inflammatory stimulation) may constitute a unique procarcinogenic microenvironment. Given these risks, close surveillance of post-nephrectomy patients is warranted. In this context, cystoscopy and ureteroscopy offer inherent advantages for detecting ureteral transitional cell carcinoma, as these modalities can identify tumors not apparent on imaging. This further highlights the importance of ureteroscopic follow-up in patients at risk for urothelial carcinoma.

## Conclusion

We report the first case of a ureteral stump tumor prolapsing into the bladder following nephrectomy. To minimize this risk, adequate resection of the distal ureter should be ensured during nephrectomy. Furthermore, for patients with a history of urothelial carcinoma—particularly those with a residual ureteral stump—follow-up surveillance should integrate cystoscopy and ureteroscopy, as these modalities can detect radiographically occult lesions.

## Supplementary Information

Additional file 1.

Additional file 2.

## Data Availability

The datasets used and/or analysed during the current study are available from the corresponding author on reasonable request.
