# Supplementary figures and images for: Long pedunculated tumor from the ureteral stump protruding into the bladder after nephrectomy: a case report
Source: Discov Oncol. 2026 May 7;17:748. doi: 10.1007/s12672-026-05028-7 (PMC13190922; doi:10.1007/s12672-026-05028-7)

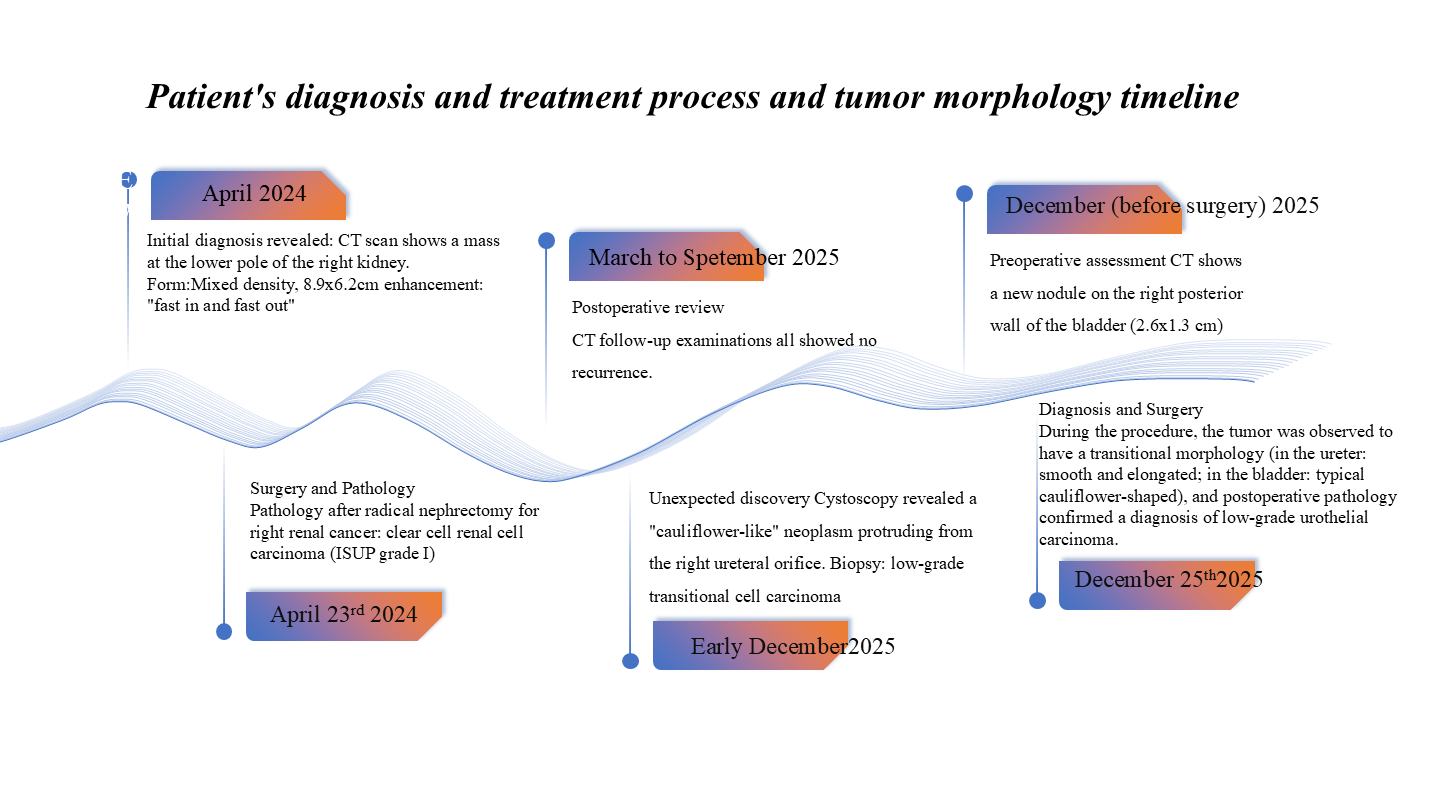

Supplement: Supplementary file 2 — Additional file 2. [file 12672_2026_5028_MOESM2_ESM.png]
